# Effect of diuretics and sodium-restricted diet on sleep apnea severity: study protocol for a randomized controlled trial

**DOI:** 10.1186/s13063-015-0699-9

**Published:** 2015-04-25

**Authors:** Cintia Zappe Fiori, Denis Martinez, Sandro Cadaval Gonçalves, Carolina Caruccio Montanari, Flavio Danni Fuchs

**Affiliations:** Graduate Studies Program in Cardiology, School of Medicine, Universidade Federal do Rio Grande do Sul, Porto Alegre, RS Brazil; Graduate Studies Program in Medical Sciences, School of Medicine, Universidade Federal do Rio Grande do Sul, Porto Alegre, RS Brazil; Cardiology Unit, Hospital de Clínicas de Porto Alegre, Porto Alegre, RS Brazil

**Keywords:** Body fluids, Diuretic, Furosemide, Sleep apnea, Sodium-restricted diet, Spironolactone, Treatment

## Abstract

**Background:**

Obstructive sleep apnea occurs as a result of increased collapsibility of the upper airway. Overnight fluid displacement from the legs to the neck causes pharyngeal narrowing and increased apnea severity. Sodium intake is associated with apnea severity. We hypothesized that interventions that decrease bodily fluid content might reduce the severity of sleep apnea.

**Methods/design:**

This is a randomized clinical trial including men with an apnea-hypopnea index greater than 30 events/hour, previously diagnosed by full-night in-laboratory polysomnography. A total of 54 men will be included and randomly assigned to three groups: Diuretic (*n* = 18), sodium-restricted diet (*n* = 18), and control (*n* = 18). The intervention will last one week. Intention-to-treat and per-protocol analyses will be performed. The diuretic group will receive combined spironolactone 100 mg plus furosemide 20 mg daily, taken in the morning. The diet group will receive a regimen with a maximum intake of 3 g of sodium per day. The control group will receive a placebo pill and will maintain all eating habits while keeping a recall diary of their dietary behavior. The primary outcome measure will be change in apnea-hypopnea index. The secondary outcome measures will be variations of: anthropometric and bioelectrical impedance variables, office blood pressure, respiratory variables from in-home level III polysomnography, excessive daytime sleepiness, glycolipid profile, C-reactive protein, 24 h urinary variables, and adverse events.

**Discussion:**

Despite the high efficacy of continuous positive airway pressure to reverse upper airway obstruction in sleep apnea, partial adherence to this form of treatment reduces its efficiency. Thus, additional forms of treating apnea need to be investigated. If the results of this proof-of-concept trial show that decreases in bodily fluid content, either by diuretic or dietary intervention, reduces the severity of sleep apnea, further investigation will be necessary before these results can be translated and adopted as an adjunct apnea therapy.

**Trial registration:**

clinicaltrials.gov NCT01945801.

## Background

Obstructive sleep apnea is a disorder characterized by repetitive collapse of the upper airway during sleep [1]. Its prevalence is increasing [2], reaching, in one study, 32% of the general adult population [3]. One reason for this growing prevalence of sleep apnea is the obesity epidemic, since obesity and neck circumference are risk factors for apnea. Sleep apnea, however, is also observed in non-obese people. In large samples, half of patients with apnea have body mass indexes below 27.6 kg/m^2^ [4].

Rostral fluid shift, that is, a shift of fluid from the lower to the upper body, produces a significant increase in neck circumference, pharyngeal resistance, and upper airway collapsibility [5-10]. Wearing elastic stockings during the day reduces the apnea-hypopnea index by more than one-third in sedentary individuals [11] and in patients with venous insufficiency [12]. Preventing fluid retention might reduce overnight fluid shift from the legs to the neck.

Diuretics given to patients with uncontrolled hypertension reduced their apnea-hypopnea index by 16%, an effect size of 0.3 [13]. In resistant hypertension, spironolactone reduced patients’ apnea-hypopnea index by approximately 50%, an effect size of 1.2 [14]. Also, diuretics improve sleep-disordered breathing and increase pharyngeal caliber in patients with severe obstructive sleep apnea and diastolic heart failure [15].

Fluid retention is proportional to sodium intake. Patients with sleep apnea have significantly greater sodium intake than patients without. In receiver operating characteristic (ROC) curve analysis, a sodium intake above a cutoff value of 2.4 g/day predicted moderate to severe sleep apnea (area under the ROC curve of 0.78) [16].

The upper limit of recommended sodium intake for healthy adults is 2.3 g/day [17]. Bibbins-Domingo et al. project that a reduction of dietary salt could halve the annual number of new cases of coronary heart disease, stroke, and myocardial infarction, and the number of deaths from any cause in the United States [18]. It is possible that part of the projected effect of salt restriction may be due to the effect of fluid retention on obstructive sleep apnea severity.

Continuous positive airway pressure (CPAP) is highly efficacious in treating obstructive sleep apnea. Many patients, however, find it difficult to tolerate its use. Poor adherence to CPAP treatment leads to reduced control of associated morbidities [19]. Reductions in blood pressure levels are seen only when CPAP use is above 5.6 hours per night [20]. Alternatives to CPAP treatment have problems of incomplete efficacy and adherence. Mandibular advancement oral appliances are similar to CPAP in controlling hypertension [21]. Various surgical approaches have diverse results [22].

Therapy of sleep apnea, therefore, is not a settled issue. Broadening comprehension of the pathogenesis of obstructive sleep apnea may help in the search for new and better tolerated treatments for the millions of people with sleep apnea.

We hypothesized that in the usual patient with apnea, without such conditions causing fluid retention as heart, renal, or venous failure, decreasing fluid retention using diuretics and a sodium-restricted diet might change sleep apnea severity. To test this hypothesis, we will conduct a trial in which the primary outcome measure is the apnea-hypopnea index. The secondary outcome measures are anthropometric and bioelectrical impedance variables, blood pressure, respiratory variables in-home polysomnography, excessive daytime sleepiness, glycolipid profile, C-reactive protein, 24 h urinary variables, and adverse events.

### Rationale

Apnea severity has been associated with overnight fluid displacement from the legs to the neck. Peripharyngeal fluid accumulation predisposes patients to upper airway collapse. Diuretics or a sodium-restricted diet offer therapeutic potential for sleep apnea by preventing fluid retention.

### Research question

Is a diuretic or a sodium-restricted diet capable of reducing the apnea-hypopnea index in comparison with a control group?

## Methods/design

### Study design

This study is a parallel randomized, blind, placebo-controlled superiority trial.

#### Eligible participants

*Inclusion criteria*

- Men aged from 18 to 60 years;
- Full-night in-laboratory polysomnography with apnea-hypopnea index greater than 30 per hour in the past three months;
- Body mass index less than 35 kg/m^2^;
- Informed consent.

*Exclusion criteria*

- Already receiving treatment for sleep apnea, including CPAP;
- Heart failure, any New York Heart Association class;
- More than 50% of the apnea events being of central origin;
- Any chronic renal disease;
- Peripheral venous or lymphatic insufficiency;
- Use of diuretics and substances with action on the central or peripheral nervous system, such as benzodiazepines, hypnotics, anticonvulsants, antidepressants, appetite suppressants, amphetamines, antiparkinson agents, muscle relaxants, bronchodilators;
- Stroke within the past 6 months or with incapacitating sequelae;
- Any physical, mental, or social condition impairing the ability to participate in the trial.

### Sample size

Sample size calculations were performed for repeated measures analysis of variance using G*Power software 3.1.9.2 [23]. Hypothesizing an effect size of 0.25 for a power of 90% at *P* alpha < 0.05, for three groups, a sample size of at least 18 in each arm of the study was required, totaling 54 individuals. In case of loss to follow-up, extra volunteers will be enrolled to compensate. Losses will be included in the intention-to-treat but not in the per-protocol analyses.

#### Randomization process

Randomly permuted blocks of six numbers, obtained from randomization.com [24], will be employed. Patients will be assigned to one of the three groups by a researcher not otherwise involved in the protocol.

### Blinding

The researchers taking measurements for either primary or secondary outcome measures, as described herein, will be blind to the group assignment. On completion of the study, volunteers will be asked to which group they believe they were assigned, to allow assessment of the blinding success [25].

The nutritionist, who delivers the diets, the only non-blinded intervention, will not be aware of the pills supplied to the unmodified diet groups and will not be involved in other steps of the protocol. The scoring of the apnea-hypopnea index will be performed remotely via the internet by a third-party certified scorer. The blinding code will be broken at the end of the study or earlier by request of regulatory committees or in the case of adverse events in connection with the group assignment.

### Recruitment

A list of patients who recently underwent polysomnography at the affiliated sleep laboratory and signed a form consenting to participate in research will be generated. Those patients with an apnea-hypopnea index greater than 30 events/hour will be selected and contacted by telephone. If they have no prescription by their physicians to start treatment in the next week, they will be invited to participate. The acceptors will be asked to visit the research center where the informed consent process will be conducted.

### Data management

The data will be entered in a password-protected computer system by researchers not otherwise involved in the protocol. The system performs a range check of the data values entered. A second person will make a visual check, comparing original documents with entered data to correct discrepancies. The database is backed up each 15 minutes in a computer located remotely at the university and in an international data safety company.

### Statistical methods

Outcomes will be analyzed by intention-to-treat and per-protocol. A low attrition rate is expected, since the interventions are of short duration and minimal risk. Missing data will be considered as missing. Data imputation will not be employed.

Normally distributed data will be presented as means and standard deviation. Non-normally distributed data will be displayed as median and interquartile range. Natural logarithm transformation of the apnea-hypopnea index in events/h will be employed to correct its non-normal distribution in analyses that assume normal data distribution. The significance of the differences between intervention groups will be tested by chi-squared test for categorical variables and generalized estimating equations method, followed by pairwise comparisons with the sequential Bonferroni correction for continuous variables. Logistic and linear regression models will be used to adjust results for age and body mass index. Results with a *P* value lower than 0.05 for alpha error will be considered statistically significant. Statistical analyses will be performed using SPSS software (SPSS Inc., Chicago, IL, USA).

### Monitoring

No committee will be monitoring the data. All analyses will be performed at the end of the study.

### Study measurements

#### Description of interventions, comparison groups, and follow-up

**Figure 1:**
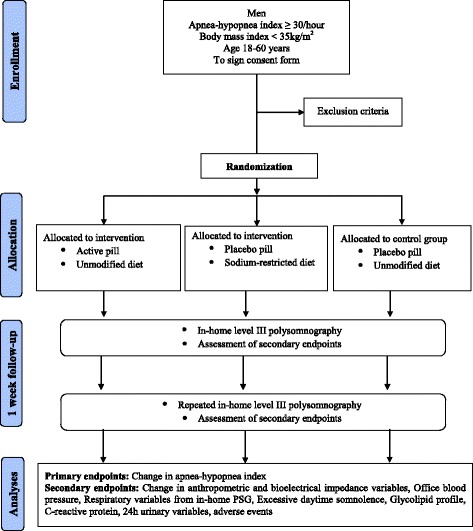
Flow diagram of the study design.

The patients will be allocated to a combination of medication and diet intervention. A detailed description of the combinations of pill plus diet interventions is shown in Figure 1.

Identical pills and flasks will contain either active drug (spironolactone 100 mg plus furosemide 20 mg) or cellulose. The diet interventions will be either instruction to maintain unchanged the present alimentary habits or a diet restricting sodium intake to a maximum of three grams per day.

The inception of the one-week intervention will occur only after volunteers confirm that in this specific week they will not participate in social events, gatherings, celebrations, travels, visits to restaurants, in such way that the adhesion to the dietary instructions will be made easier.

The sodium-restricted diet will enforce the following rules: (1) Do not use salt in cooking; (2) Do not use the salt shaker; (3) To flavor foods, use spices, herbs, and other seasonings, such as olive oil, lemon, herbs, garlic, onion, parsley and chives, instead of salt; (4) Do not eat any industrialized food, such as sauces, soups, sausages, canned food, frozen, salted snacks, cheese, salami or sausages; (5) Eat fresh salads and vegetables; (6) Do not add high-sodium seasonings, such as soy sauce; (7) Eat fruit for dessert; do not eat baked desserts. The complete dietary rules will be delivered to each volunteer in a four-page handout.

### Ethics

The study was approved by the Ethics Committee of the Hospital de Clínicas de Porto Alegre under the number 130272. This committee is accredited by the Office of Human Research Protection as an Institutional Review Board (IRB0000921). The study has been registered under the number NCT01945801 at www.clinicaltrials.gov. All participants will be asked to sign the approved informed consent form prior to participation in the study.

Authorship eligibility will follow the guidelines of the International Committee of Medical Journal Editors. Professional writers will not be used. The trial results will be published in a high impact international journal. The investigators will communicate trial results to participants via email. No publication restrictions will be implemented.

#### Outcomes

*Primary outcomes*

- Number of apneas and hypopneas per hour of sleep (apnea-hypopnea index)

*Secondary outcomes*

- Adverse events
- Anthropometry (body weight and height; neck, waist, hip, ankle, and calf circumferences)
- Bioelectrical impedance variables
- Urinary electrolytes and metabolites
- Office blood pressure
- Respiratory variables from the type III portable polysomnography
- Excessive daytime somnolence
- Glycolipid profile
- Inflammatory marker

### Follow-up and duration of the study

There will be two visits at the clinical research center, one before (baseline) and one after (follow-up) the one-week pill plus diet intervention.

### Assessment of outcomes

#### Sleep respiratory variables

The apnea-hypopnea index in events/hour will be measured at baseline and follow-up nights by type III portable polysomnography. Minimum oxygen saturation will also be analyzed. The equipment employed in the study (SOMNOcheck effort, Weinmann GmbH, Hamburg, Germany) was validated by our research group and showed diagnostic performance similar to in-laboratory attended polysomnography [26].

### Adverse events

The adverse events will be investigated by open questions, including general symptoms, and by specific questions regarding symptoms potentially related with the drugs used in the trial, as dizziness, cramps, dehydration, and hypotension. The rate of adverse effects will be determined by comparing the frequency of adverse events in the drug arm with in the placebo arm.

### Bioelectrical impedance

The body composition will be determined in the morning, while fasting, with a tetrapolar bioelectrical impedance device (Biodynamics, model 450, WA, USA) [27,28], at baseline and follow-up visits. The measurements will be made after the blood sample collection, in fasting, without ingesting any liquid.

### Blood pressure

The average office blood pressure will be measured according to the American Heart Association guidelines [29]. The same validated oscillometric device (OMRON CP-705) [30] will be employed at baseline and follow-up visits.

### Excessive daytime sleepiness

The volunteers will complete the Epworth sleepiness scale [31] to evaluate somnolence. The same model of form will be presented at baseline and follow-up visits.

### Biochemical evaluation

The collection of blood samples for biochemical evaluation will be performed in all subjects at baseline and follow-up visits. Enrollees will be asked to arrive at the center early in the morning in fasting. Blood samples will be drawn directly in five different sampling tubes. The samples will be rushed to the lab to measure plasma levels of: aldosterone, renin, ultrasensitive C-reactive protein, cholesterol total, high density lipoprotein-cholesterol, triglycerides, and glucose. At each visit the individuals will return the refrigerated container previously handed to them for 24-hour urinary collection. Dosages of urinary aldosterone, sodium, potassium, urea, and creatinine will be obtained.

## Discussion

This experiment is designed to test the hypothesis that abating fluid retention may affect apnea severity. In the drug arm, the intervention will be an association of a loop diuretic with a potassium-sparing aldosterone-antagonist agent in moderate doses, to ensure a negative balance of sodium and water. In the diet arm, a strict low-sodium diet will be implemented. To serve as control, the trial will have a placebo arm.

Besides the effect on the apnea-hypopnea index, the study will measure potential mediators and consequences of these interventions. If the hypothesis regarding fluid retention is confirmed, further trials will be necessary to verify whether the effect is due to fluid shift from the legs to the neck. For this, measurement of overnight fluid shift by bioelectrical impedance will be necessary.

The therapeutic efficiency and efficacy of diuretic and diet interventions will not be tested. It will remain to be confirmed whether the effect is maintained for years and if it is attainable with lower doses of diuretics and with diets less restricted in sodium, that is, more agreeable to the patients. Clinical trials with large samples and long term follow-up will be necessary to clarify these questions.

## Trial status

At the time of manuscript submission, the enrollment of volunteers is ongoing.

## Abbreviations

CPAP: Continuous positive airway pressure
ROC: Receiver operating characteristic
